# Maternal Exposure to Ambient Ozone and Fetal Critical Congenital Heart Disease in China: A Large Multicenter Retrospective Cohort Study

**DOI:** 10.3390/toxics13060463

**Published:** 2025-05-31

**Authors:** Yanping Ruan, Yaqi Wang, Zhiyong Zou, Jing Li, Yihua He

**Affiliations:** 1 Echocardiography Medical Center, Maternal-Fetal Medicine Center in Fetal Heart Disease, Beijing Anzhen Hospital, Capital Medical University, Beijing 100029, China; yanping.ruan@163.com; 2 Institute of Child and Adolescent Health, School of Public Health, Peking University, Beijing 100191, China; 2211110227@bjmu.edu.cn (Y.W.); harveyzou2002@bjmu.edu.cn (Z.Z.); 3 National Health Commission Key Laboratory of Reproductive Health, Beijing 100191, China

**Keywords:** congenital heart defects, critical congenital heart defects, air pollution, ozone, periconceptional period

## Abstract

The relevance of O_3_ exposure in critical congenital heart disease (CCHD) remains uncertain and requires further investigation. The present study aims at quantitatively assessing the association between ambient O_3_ exposure during the early pregnancy period with fetal CCHD and identifying possible susceptible exposure windows. A retrospective cohort study involving 24,516 pregnant women was conducted using data from the Maternal–Fetal Medicine Consultation Network, which encompassed 1313 medical centers across China from 2013 to 2021. We extracted daily O_3_ concentrations from a validated grid dataset with a spatial resolution of 0.1° at each participant’s residential county to assess ambient O_3_ exposure, followed by calculating the average exposure levels in the periconceptional period, embryonic period, first trimester, and preconception period. The diagnosis of CCHD was based on fetal echocardiography. Exposure–response analyses were carried out using logistic regression models. During the study period, a total of 1541 (17.4%) subjects were diagnosed with fetal CCHD. Each 10 µg/m^3^ increase in ambient O_3_ exposure in the periconceptional period was associated with a 26.0% increase in the odds of CCHD (odds ratio [OR]: 1.260, 95% confidence interval [CI]: 1.189, 1.335; *p* < 0.001). Importantly, the association was not modified by factors including maternal age and occupation status, paternal age and smoking status, conception mode, and the presence of risk factors. In the sensitivity analysis, significant associations were observed between O_3_ exposure and CCHD in the embryonic period, first trimester, and preconception period, which was consistent with the results of the main analyses. These findings suggest that lowering ambient O_3_ exposure in the preconception and early pregnancy periods may be beneficial in reducing the risk of fetal CCHD, especially in regions with elevated O_3_ levels.

## 1. Introduction

Congenital heart disease (CHD) is a major cause of congenital-anomaly-related infant mortality and has emerged as a critical global issue, particularly in low- and middle-income countries [1]. Cyanotic CHD, often denoted as critical congenital heart disease (CCHD), constitutes a noteworthy subset of CHD [2] and accounts for 20% of neonatal mortality [3]. CCHD infants require surgical intervention or other necessary procedures within their first year of life (FYOL) [4]. According to the Global Burden of CHD in 2019, CHD affected 9 out of every 1000 live births globally, and approximately 25% cases of CHD were considered to be CCHD [5]. China presents an average CHD prevalence of about 8.5 per 1000 live births, with the CCHD prevalence estimated to be 1.4 per 1000 live births [6]. CCHD can lead to significantly higher incidence and death rates if not identified promptly. Timely recognition and treatment can significantly mitigate the risk of fatality or severe complications [7]. Consequently, this not only enhances the quality of life for patients, but also reduces the economic burden associated with these conditions. Advancements in ultrasonographic technology have contributed to a steady improvement in CCHD identification in prenatal testing in the past two decades [8,9,10]. This improvement enhances the perioperative clinical status pertaining to neonates affected by CCHD [11,12]. However, the etiology of CCHD is still largely unknown [2]. The identification and management of modifiable risk factors continue to be the key focus in the prevention and control of fetal CCHD.

According to the Developmental Origins of Health and Disease (DOHaD) hypothesis, ambient exposures in people’s early life significantly impact health outcomes [13]. While according to previous studies, mothers being exposed to air pollution during pregnancy may have potential impacts on specific subtypes of CCHD, these results are inconsistent. A systematic review found a positive association between carbon monoxide (CO) exposure and the odds of Tetralogy of Fallot (TOF) yet failed to find significant associations between fine particulate matter (PM_2.5_), nitrogen dioxide (NO_2_), and conditions including TOF, Transposition of the Great Arteries (TGA), and Coarctation of the Aorta (COA) [14]. Consequently, future research is suggested to clarify the association between air pollution and CCHD.

Along with global climate change, ozone (O_3_) has demonstrated an aggravated uptrend in recent years, making it a primary public health concern globally [15]. Several mechanistic studies found that gestational exposure to O_3_ enhanced placental oxidative stress and inflammation, altered neural cell migration, caused outflow tract defects, and affected the placenta’s function, thereby being involved in CHD [16,17,18]. Additionally, according to epidemiological evidence, maternal exposure to O_3_ significantly increased the odds of CHD [19,20,21,22]. However, existing research has not clearly elucidated the association between the two. Moreover, most previous studies exploring impacts of maternal O_3_ exposure on CHD were from developed countries [19,23,24,25,26], which have a lower O_3_ concentration compared to China and other areas with heavy pollution. Notably, findings within these studies have been inconsistent. While one study identified an obvious association between maternal O_3_ exposure and higher odds of CHD [26], others did not observe a significant association [19,23,24,25]. Such differences in association may be affected by the variable distribution of O_3_ levels. Hence, further investigations are warranted, particularly in regions with varying O_3_ exposure ranges.

In previous studies, the majority focused on relatively short-term exposure to O_3_ during 3–8 weeks of gestation (the embryonic period), a key period for fetus cardiac development [27]. However, recent advancements in early fetal cardiac imaging have demonstrated that cardiac refinement and maturation extend into the 10th to 12th weeks of gestation (Embryology Heart Historic Movie 4). This suggests that the entire first trimester may be a period of vulnerability. Besides, the crucial windows are not always in line with the cardiac development time of the fetus, and high-concentration pollutants accumulating in the long term may affect the relevance of air pollution to CHD [14,28]. Research has not clearly elucidated how long-term O_3_ exposure affects CCHD development, which is a knowledge gap with substantial public health as well as clinical significance. The periconceptional period, defined as the three months before conception through the thirteenth week of gestation, is a pivotal period sensitive to environmental exposure due to the epigenetic reprogramming of the developing fetus [29]. This period includes the preconception period, featuring preantral follicle maturation and ovulation, which lay the foundation for cardiac development [30,31], and the first trimester, which significantly affects fetal organ development and neural tube formation [32]. While periconceptional exposure to fine particulate matter is reported to be associated with CCHD in some studies [28], the exact impacts of O_3_ exposure in this period remain less certain and warrant further investigation.

Utilizing data from the Maternal–Fetal Medicine Consultation Network of the National Clinical Research Center for Cardiovascular Diseases that covered 1313 medical centers in China in the period of 2013–2021, we conducted a nationwide retrospective cohort study to quantitatively examine the relevance of maternal exposure to O_3_ during periconception to the risk of CCHD.

## 2. Materials and Methods

### 2.1. Study Design and Participants

The study initially enrolled 27,817 pregnant women, initially screened at 1313 medical centers across China. Figure S1 presents the location of the medical centers. Among them, those who exhibited high-risk factors for CHD and presented common indications of referral for fetal echocardiography (FECG) (the American Heart Association, AHA) [11] were referred to the Maternal–Fetal Medicine Center for Fetal Heart Disease at Beijing Anzhen Hospital. There, they underwent FECG for a definitive diagnosis, with all relevant data collected by the Maternal–Fetal Medicine Center of AnZhen hospital. Figure S1 illustrates the distribution of referral hospitals. Several criteria were adopted for identifying pregnant women with high fetal CHD risk: the presence of maternal metabolic diseases (gestational diabetes mellitus [GDM] and uncontrolled phenylketonuria [PKU]), maternal first-trimester infection (rubella virus), family history of CHD, suspected structural cardiac abnormalities detected during obstetric ultrasound, irregular fetal rhythm, fetal bradycardia, fetal karyotype abnormalities, monochorionic twinning, or other indications recommended by the AHA [11].

We selected pregnant women with fetuses diagnosed with CCHD and a control group of pregnant women with fetuses exhibiting no cardiac defects as subjects. The following women were excluded: (1) 133 with missing data on age; (2) 144 lacking data on gestation week at examination; (3) 393 with duplicated medical records; (4) 2380 fetuses without CCHD but with other types of CHD; yielding 24,516 pregnant women after exclusions. Figure S2 displays the participant selection flowchart.

### 2.2. Ascertainment and Classification of CCHD

Fetal echocardiography (F-Echo) was adopted for the diagnosis of CCHD with the assistance of a Voluson E8-RAB4-8 machine (GE Healthcare, Little Chalfont, UK). FECG images were acquired following the requirements of the AHA [11] and ISUOG [9]. Highly skilled associate chief physicians and chief physicians took charge of the F-Echo, utilizing grayscale, color images, and pulsed wave Doppler. Diagnosis was conducted via a comprehensive screening process, encompassing multiple sections, including assessment of the four-chamber view, left and right ventricular outflow tracts (LVOT and RVOT), three-vessel (3V) view, three-vessels and trachea (3VT) view, and sagittal views of the superior and inferior vena cava, aortic arch, and ductal arch. FECG findings were compared with autopsy findings, verifying the high accuracy of the examination in diagnosing CHD. FECG achieved a diagnostic coincidence rate of 98.8% for critical cardiac abnormalities [33].

CCHD refers to cardiac structural malformations for which neonates require early surgical or catheter intervention in the FYOL to survive [34]. Typical types of CCHD include Tetralogy of Fallot, aortic coarctation, double-outlet right ventricle, total anomalous pulmonary venous connection (TAPVC), interrupted aortic arch, hypoplastic left heart syndrome, D-transposition of the great arteries, single ventricle, pulmonary atresia with intact septum, tricuspid atresia, truncus arteriosus, and Ebstein anomaly [35,36].

### 2.3. Exposure Assessment

Based on the Tracking Air Pollution (TAP) data in China datasets (available at http://tapdata.org.cn/ [accessed on 31 May 2024]), we extracted daily maximum 8 h average (MDA8) O_3_ grid data (spatial resolution: 0.1°, approximately 11 km × 11 km at China’s latitudes) in China from 2013 to 2021. The TAP database used the three-stage model for forecasting O_3_ levels across China for the period 2013–2020. This model integrated ground measurements under the reference state, CMAQ simulations, Ozone Monitoring Instrument (OMI) satellite O_3_ profiles (PROFOZ; v0.9.3, level 2), MERRA-2 meteorology parameters, the MODIS Normalized Difference Vegetation Index (NDVI), and National Centers for Environmental Information (NCEI) annual night light data. The 5-fold cross-validation results of the three-stage O_3_ prediction model yielded an R^2^ of 0.84 compared to ground-based measurements [37].

The daily MDA8 O_3_ concentrations specific to each subject’s geocoded address were extracted for assessing individual exposure to O_3_. The available residential address for each subject was at the county level. Then the average exposure level in the periconceptional period was calculated, together with an assessment of exposure to O_3_ in (1) the embryonic (3–8 weeks of gestation) period; (2) the first-trimester (1–13 weeks of gestation) period; and (3) the preconception (3 months before the last menstrual period (LMP)) period, which were considered critical time periods for ascertaining the causality between maternal risk factors and adverse pregnancy outcomes [28,38]. The gestational week started on the 1st day of the LMP.

### 2.4. Covariate Assessment

The study collected demographic information, lifestyle factors, and medical history data pertaining to both subjects and their spouses using a concise questionnaire. Demographic and lifestyle information were age, occupation, smoking status, alcohol consumption, exposure to radioactive substances, pet keeping, and home decoration practices, recorded within six months before pregnancy. Medication history encompassed diseases such as DM, GDM, upper respiratory infections (URI) within 3 months before conception, hemorrhage and medication history in the first trimester, PKU, thyroid disease, metabolic disorders, and connective tissue diseases, family history of fetal CHD, adverse prenatal genetic test results, and gestational psychological stress. Risk factors were the presence of any of the aforementioned medical history as well as having pets or decorations in the household within the 3 months before conception. Clinical information, such as gestational age in weeks, number of fetuses, and conception mode, was extracted from electronic medical records.

Grid data on daily 24 h mean PM_2.5_ and NO_2_ concentrations (with spatial resolutions of 1 km × 1 km and 10 km × 10 km, respectively) were obtained from the previously validated ChinaHighAirPollutants (CHAP) dataset (available at https://weijing-rs.github.io/product.html [accessed on 31 May 2024]) [39,40,41]. We obtained 24 h mean grid data on temperature (°K) and relative humidity (%) in China during the period of 2013–2021 from the 5th generation of the European Re-Analysis (ERA5)-Land re-analysis dataset (spatial resolution: 9 km × 9 km) [42]. For each subject, the available residential address was at the county level. Calculating the average values of all grids within each corresponding county’s geographical boundary yielded daily exposure estimates in °K and %. We further calculated the average temperature and relative humidity levels for the critical time periods.

### 2.5. Statistical Analysis

We adopted logistic regression models for examining the impact of maternal O_3_ exposure during the periconceptional period on CHD, including the exposure as a continuous variable, thereby estimating the ORs and their 95% CIs for CHD associated with each 10 μg/m^3^ increase in exposure to O_3_. Different sets of covariates were adjusted during the analysis, explaining the potential confounders. Three models were constructed: Model 1 (adjusting for maternal age, maternal occupation status, paternal age, average temperature [natural cubic splines with 3 degrees of freedom] and relative humidity [natural cubic splines with 3 degrees of freedom]); Model 2 (additionally accounted for paternal lifestyle factors including smoking status and alcohol consumption); and Model 3 (further adjusting for clinical factors including gestational age, conception mode, fetus number, presence of risk factors, and season of conception). In the categorical analysis, the distribution of exposure levels for all observations was divided into Quartiles 1–4, with the ORs of Quartile 2–4 versus Quartile 1 being estimated. The respective median was included as a continuous variable, aiming at testing the linear trends with regard to the quartiles. We also paid attention to the association between maternal exposure to O_3_ and the top five most common subtypes of CCHD. Additionally, we conducted stratified analysis by maternal age, occupation status, paternal age, smoking status, conception mode, and the presence of risk factors. Likelihood ratio tests were used for examining the potential effect modifications. Sensitivity analyses were conducted to assess the robustness of the results. In an extended version of Model 3, we further adjusted for PM_2.5_ and NO_2_ exposure during the periconceptional period. Additionally, we examined ambient O_3_ exposure in separate models corresponding to the three critical time windows to better elucidate its association with CHD. Data analysis relied on R version 4.2.2 [43]. A two-sided *p* < 0.05 reported statistical significance.

## 3. Results

### 3.1. Subject Characteristics

**Figure 1:**
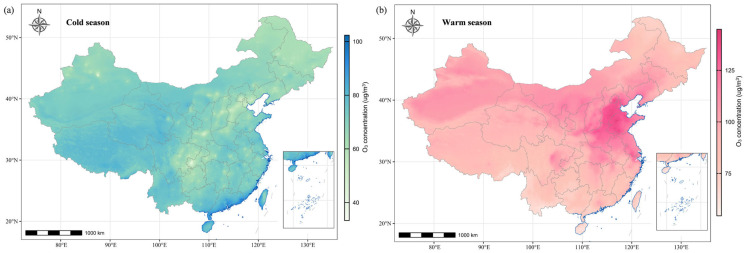
The spatial distributions of average O_3_ concentrations for the period of 2013–2021. (**a**) The O_3_ concentrations (unit: µg/m^3^) during the cold season refer to the average of daily mean concentrations from October through March of the subsequent year. (**b**) The O_3_ concentrations (unit: µg/m^3^) during the warm season refer to the average of daily mean concentrations from April through September.

Table 1 presents the characteristics of 24,516 subjects from the Maternal–Fetal Medicine Consultation Network. Among all the subjects, 1541 (6.3%) were diagnosed with CCHD, and the top five most common types of CCHD were Tetralogy of Fallot (*n* = 426 [27.6%]), aortic coarctation (n = 250 [16.2%]), double-outlet right ventricle (n = 231 [15.0%]), D-tansposition of the great arteries (n = 163 [10.6%]), and TAPVC (n = 99 [6.4%]) (Table S1). The subjects and their spouses had a mean age of 31.0 years and 32.6 years, respectively, and 19,341 (78.9%) of subjects were under 35 years old. A total of 17,119 (69.8%) subjects were referred to Beijing Anzhen hospital, and 21,205 (86.5%) women underwent FECG between 15 and 28 weeks of gestation. Women in the CCHD group were more likely to be unemployed and their spouses showed a larger likelihood of having a history of drinking and smoking (*p* < 0.001). Figure 1 shows the spatial distribution pertaining to seasonal average O_3_ exposure levels during the period of 2013–2021 in China. The high-pollution areas were concentrated in the northeast of China. All subjects had an average O_3_ exposure during the periconceptional period of 93.9 µg/m^3^ (standard deviation [SD]: 29.9 µg/m^3^), which was in the range of 38.1–161.6 µg/m^3^. The average O_3_ exposures of the three critical time periods were 95.6 µg/m^3^ (SD: 43.3 µg/m^3^), 95.9 µg/m³ (SD: 40.2 µg/m^3^), and 92.0 µg/m^3^ (SD: 39.4 µg/m^3^), respectively.

### 3.2. Exposure–Response Analysis

Table 2 and Table S5 show the estimated odds ratios for CCHD associated with maternal O_3_ exposure during the periconceptional period. Overall, with each 10 μg/m^3^ increment in O_3_ exposure during the periconceptional period, the estimated OR of CCHD was 1.406 (95% CI: 1.330, 1.486) in Model 1 (Table S5), 1.422 (95% CI: 1.345, 1.503) in Model 2 (Table S5), and 1.260 (95% CI: 1.189, 1.335) in Model 3 (Table 2), respectively. In categorical analysis, we observed significantly and monotonically elevated ORs across quartiles of exposure to O_3_ during the periconceptional period (*p* values for linear trend < 0.05, Table 2). For the five most common subtypes of CCHD, maternal exposure to O_3_ did not exhibit a significant association with TOF, COA, DORV, TGA, or TAPVC. Complete subtype analyses are provided in Supplementary Table S4.

### 3.3. Stratified and Sensitivity Analyses

**Figure 2:**
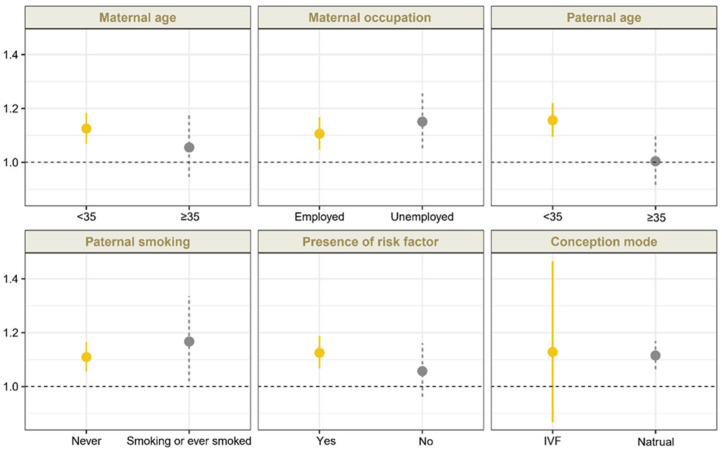
A stratified analysis of the association between maternal O_3_ exposure during periconceptional period and CCHD (by maternal age and occupation status, paternal age and smoking status, presence of risk factors, and conception mode). Risk factors included the following: commodities (DM, GDM, URI within 3 months before conception, hemorrhage during the first trimester, PKU, metabolic disorders, connective tissue diseases, and thyroid disease), drug use during the first trimester, history of CHD, adverse results of prenatal genetic test results, radioactive substances exposure, mental stress, and household environmental factors (keeping pets, decoration) during early pregnancy. The presence of any of the aforementioned medical history was defined as having a risk factor. ORs and 95% CIs were estimated per 10 ug/m^3^ increase in O_3_ exposure.

Sensitivity analyses confirmed that the association between O_3_ exposure during the periconceptional period and CCHD remained robust after adjustment for ambient PM_2.5_ and NO_2_ exposure, although the effect estimates were slightly attenuated (Table 2). For exposure windows, we found significant associations between exposure to O_3_ and CCHD during the embryonic period (OR 1.136 [95% CI, 1.090 to 1.184]), the first trimester (OR, 1.181 [95% CI, 1.128 to 1.237]), and the preconception period (OR, 1.117 [95% CI, 1.058 to 1.181]), which were consistent with results of the main analyses (Table 3). Moreover, the ORs for CCHD exhibited a monotonic increase with O_3_ exposure at relatively lower levels and a slight attenuation at higher exposure levels during these three periods (Table 3). For the subtypes of CCHD, we also found significant associations between maternal O_3_ exposure and TOF, COA, TGA, and TAPVC during these three periods. Figure 2 demonstrates the associations between O_3_ exposure during the periconceptional period and CCHD by stratified analysis (Table S3 in Supplementary Materials). No significant effect modification by the aforementioned factors was identified (*p* > 0.05).

## 4. Discussion

A retrospective cohort study was conducted on 24,516 pregnant women from 1313 medical centers in China from 2013 to 2021, confirming a linear exposure–response association between periconceptional exposure to ambient O_3_, ranging from 38.1 to 161.6 µg/m^3^, and increased odds of CCHD, which was unlikely to be modified by patient characteristics included in the stratified analysis.

Among the several previous studies examining the association of maternal O_3_ exposure with overall CHD, most demonstrated that exposure to O_3_ during the embryonic period and the first trimester increased the odds of CHD [20,23,25,44]. However, research has not quantitatively elucidated the relevance of maternal O_3_ exposure to CCHD, considering the small proportion of fetuses with CCHD. In China, CHDs exhibit a mean prevalence of about 8.5 per 1000 live births [6], and only 25% babies born with a CHD has a CCHD [4]. The insufficient sample size of fetuses with CCHD may hinder studies from achieving statistical power. In addition, our estimates for CCHD during the periconceptional period (OR: 1.260 [95% CI: 1.189–1.335]), the embryonic period (OR: 1.136 [95% CI: 1.090–1.184]), and the first trimester (OR: 1.181 [95% CI: 1.128–1.237]) were a little higher than estimates for overall CHD in previous studies (ORs range: 1.02–1.16). Several possible reasons may account for this inconsistency: Firstly, fetuses with CCHD may have a relatively higher level of inflammation compared to those with other types of CHD [2], which could make them more vulnerable to air pollution exposure. Secondly, the average O_3_ exposure in the four periods in our study was over 90.0 µg/m^3^, surpassing that of most previous studies (26.5 µg/m^3^ to 72.4 µg/m^3^). It is plausible that the estimates could potentially be higher in regions with higher pollution levels. Previous studies also reported that fetuses with maternal exposure to PM_2.5_ and NO_2_ showed higher odds of TGA, a common type of CCHD [45,46]. According to the spatial PM_2.5_ and NO_2_ concentration distribution from the CHAP dataset, areas with elevated O_3_ concentrations often coincide with increased PM_2.5_ and NO_2_ [41,47]. This coexistence of multiple pollutants raises concerns regarding potential data bias. To address potential bias, we performed separate adjustments for PM_2.5_ and NO_2_ exposure while examining the relevance of O_3_ to CCHD, and the findings were consistent with the main analysis, reinforcing the robustness of the results.

In previous studies, the embryonic period and the first trimester have been identified as critical exposure windows for the majority of teratogenic agents and CHD, during which cardiac development occurs [27]. Nevertheless, the association of air pollution with CHD involves high-concentration pollutants or relevant metabolites accumulating over the long term, rather than high-level acute exposure [28]. In this study, we observed the obvious relevance of O_3_ exposure during the preconception period and periconceptional period to higher odds of CCHD, demonstrating that heightened awareness of reducing O_3_ exposure is needed not only during the first trimester but also in earlier and longer periods, specifically 3 months before conception. Previous studies have rarely paid attention to how air pollution affects CHD during the preconception period. To date, only one study used a nationwide surveillance-based case-control design and examined the association between maternal exposure to PM_2.5_ during preconception and risk of CHD in offspring [28]. They found that PM_2.5_ exposure during preconception was statistically associated with higher odds of overall CHD, but they did not find its association with conotruncal defects, a subtype of CCHD [28]. The impact of O_3_ exposure on CCHD was not explored in this study. In addition, the study design and selection of the population of this study differed from our study. In this study, they used the National Population-Based Birth Defects Surveillance System, identifying birth defects from 28 weeks of gestation to 42 days after birth. In this case, some aborted fetuses were not monitored or included, introducing a survival bias. In our study, 86% of the study participants were diagnosed with CHD before 28 weeks and were enrolled regardless of whether or not there were abortions or stillbirths, which somewhat reduces the influence of survival bias on our results.

Several mechanistic studies indicate that either of the parents being exposed to air pollution during preconception, or the early stages of pregnancy, can have negative impacts on sperm and ova gametogenesis, possibly reducing the ovarian reserve [48], disrupting the blastocyst-stage cell lineage segregation [49], and detrimentally affecting fetal and neonatal development in the long term [50,51]. According to in vitro and in vivo toxicological studies, such exposure is capable of impacting the gametes’ epigenetic signatures and DNA methylation modes, raising maternal oxidative stress, damaging endocrine function in the process of gametogenesis, and changing placental mitochondrial DNA content [52,53,54]. Similarly, epidemiological data also confirm the relevance of pre-pregnancy maternal exposure to air pollutants to health outcomes, represented by birth defects [55]. CHD is a representative congenital anomaly and can also be affected by air pollution during preconception, despite the limited evidence for such an association. With the aim of clarifying how air pollution affects CHD, prospective cohort studies, RCTs (which involve indoor air purification, the use of N95 masks, and other interventions), etc., should be thoroughly conducted in the future.

This study has several strengths. First, data used here came from the National Clinical Research Center for Cardiovascular Diseases, which is equipped with authentic maternal–fetal records and a massive number of fetal CCHD cases (n = 1541). Second, the large sample size of 24,516 pregnant women and a wide geographic scope assisted us in statistically testing the relevance of ambient O_3_ to CCHD, especially at high exposure levels, and more deeply examining the potentially critical exposure windows.

There are also several limitations. First, most of the subjects were pregnant women referred to Beijing Anzhen hospital. The majority of these participants presented with more risk factors for CHD than the general population, which may have led us to overestimate the association between maternal exposure to O_3_ and CCHD, and limited our results from being generalized to the general population. Future studies are suggested to include a broader spectrum of participants drawn from diverse medical centers across multiple geographical regions. Second, we used county-specific O_3_ for assigning individual exposure specific to all subjects during pregnancy. Information on exact residential address, maternal activity patterns, and residential mobility during pregnancy were deficient, leading to exposure misclassification. Third, we adjusted for a number of risk factors but cannot rule out residual confounding from unmeasured risk factors (e.g., physical activity) due to the observational design. Finally, fetal sex information was unavailable due to ethical regulations prohibiting its disclosure or documentation prior to birth. In addition, the absence of linkage to postnatal records prevented retrospective identification. As a result, sex-specific analyses could not be performed, despite established differences in CCHD susceptibility between sexes. Future studies should include fetal sex as a key variable to better elucidate sex-specific associations.

## 5. Conclusions

In conclusion, maternal O_3_ exposure in the periconceptional period can induce higher odds of CCHD in offspring. Hence, reducing ambient O_3_ exposure in the preconception period and during early pregnancy can prevent women from fetal CCHD, particularly in areas where the ambient O_3_ concentration is high. Future studies are suggested to generalize the findings in this study by including other populations and to elucidate the relevant biological mechanisms.

## Figures and Tables

**Table 1:** Characteristics of the study subjects in China, 2013–2021.

Characteristics	Total (N = 24,516)	CCHD (N = 1541)	No CHD (N = 22,975)	*p* Value
Maternal factors				
Maternal age, years	31.0 (4.2)	30.5 (4.6)	31.1 (4.2)	<0.001
Maternal occupation status, n (%)				<0.001
Employed	19,633 (80.1)	1074 (69.7)	18,559 (80.8)	
Unemployed	4055 (16.5)	430 (27.9)	3625 (15.8)	
Unknown	828 (3.4)	37 (2.4)	791 (3.4)	
Paternal factors				
Paternal age, years	32.6 (5.1)	31.7 (5.2)	32.7 (5.0)	<0.001
Smoking status, n (%)				<0.001
Smoking or ever smoked	2139 (8.7)	205 (13.3)	1934 (8.4)	
Never	22,377 (91.3)	1336 (86.7)	21,041 (91.6)	
Alcohol consumption, n (%)				<0.001
Drinking or ever drank	989 (4.0)	112 (7.3)	877 (3.8)	
Never	23,527 (96.0)	1429 (92.7)	22,098 (96.2)	
Clinical factors				
Gestational week at time of fetal echocardiography ^a^, n (%)				<0.001
15–28	21,205 (86.5)	1281 (83.1)	19,924 (86.7)	
29–40	3311 (13.5)	260 (16.9)	3051 (13.3)	
Conception method, n (%)				0.615
Natural	23,591 (96.2)	1487 (96.5)	22,104 (96.2)	
IVF	925 (3.8)	54 (3.5)	871 (3.8)	
Fetus number, n (%)				0.005
Singleton	24,027 (98.0)	1495 (97.0)	22,532 (98.1)	
Multiple	489 (2.0)	46 (3.0)	443 (1.9)	
Presence of risk factors ^b^, n (%)				<0.001
Yes	16,681 (68.0)	1135 (73.7)	15,546 (67.7)	
No	7835 (32.0)	406 (26.3)	7429 (32.3)	
Conception season ^c^, n (%)				0.996
Warm	12,018 (49.0)	756 (49.1)	11,262 (49.0)	
Cold	12,498 (51.0)	785 (50.9)	11,713 (51.0)	
O_3_ exposure, μg/m^3^				
Periconceptional period	93.9 (29.9)	92.3 (27.7)	94.0 (30.0)	0.036
Embryonic period	95.6 (43.3)	93.9 (40.1)	95.7 (43.5)	0.138
The first trimester	95.9 (40.2)	94.2 (37.1)	96.0 (40.4)	0.090
Preconception period	92.1 (39.4)	90.7 (35.9)	92.2 (39.7)	0.140

Values are presented as mean ± SD or n (%). CHD, Congenital heart defects; CCHD, critical congenital heart defects; SD, standard deviation; GW, gestational week; IVF, in vitro fertilization. ^a^ Gestational week represents the age of the fetus at the time when a pregnant woman underwent a FECG. ^b^ Risk factors included the following: commodities (DM, GDM, URI within 3 months before conception, hemorrhage during the first trimester, PKU, metabolic disorders, connective tissue diseases, and thyroid disease), drug use during the first trimester, history of CHD, adverse prenatal genetic test results, radioactive substance exposure, mental stress, and household environmental factors (keeping pets, decorations) during early pregnancy. The presence of any of the aforementioned medical history was defined as having a risk factor. ^c^ The warm season was from April to September; the cold season was from October to December, and January to March.

**Table 2:** Association between exposure to ambient O_3_ during periconceptional period ^a^ and CCHD assessed by single- and 2-pollutant models.

O_3_ Exposure	ORs (95% CIs) ^b^		
	Single ^c^	Adjusted for PM_2.5_^d^	Adjusted for NO_2_^e^
Per 10 ug/m^3^	1.260 (1.189, 1.335)	1.372 (1.289, 1.461)	1.255 (1.168, 1.348)
Quartile 1	Ref	Ref	Ref
Quartile 2	1.381 (1.170, 1.629)	1.338 (1.085, 1.650)	1.096 (0.874, 1.374)
Quartile 3	1.511 (1.151, 1.983)	1.824 (1.304, 2.552)	1.406 (0.979, 2.018)
Quartile 4	1.803 (1.293, 2.514)	1.685 (1.115, 2.546)	1.267 (0.818, 1.965)
*p* for linear trend ^f^	<0.001	<0.05	0.330

CCHD, critical congenital heart defects; NO_2_, nitrogen dioxide; O_3_, ozone; PM_2.5_, particulate matter ≤ 2.5 μm or less in diameter; OR, odds ratio; CI, confidence interval. ^a^ Periconceptional period was defined as 3 months before LMP until 13 weeks of gestation. ^b^ Logistic regression models were constructed for estimating ORs and 95% CIs. ^c^ Model was adjusted for maternal age and occupation status, paternal age, average temperature (natural cubic splines with 3 degrees of freedom [df]), relative humidity (natural cubic splines with 3 df), paternal smoking status, alcohol consumption, gestational age, conception mode, fetus number, presence of risk factors, and season of conception, as specified in Model 3. ^d^ Model was further adjusted for PM_2.5_ exposure in periconceptional period based on Model 3. ^e^ Model was further adjusted for NO_2_ exposure in periconceptional period based on Model 3. ^f^ *p* for linear trend was tested based on continuous variable in model.

**Table 3:** Association between exposure to ambient O_3_ and CCHD in China, 2013–2021.

O_3_ Exposure	ORs (95% CIs) ^a^		
	Embryonic Period ^b^	The First Trimester ^c^	Preconception Period ^d^
Per 10 ug/m^3^	1.136 (1.090, 1.184)	1.181 (1.128, 1.237)	1.117 (1.058, 1.181)
Quartile 1	Ref	Ref	Ref
Quartile 2	1.332 (1.123, 1.579)	1.456 (1.220, 1.737)	1.297 (1.096, 1.536)
Quartile 3	2.472 (1.826, 3.347)	2.947 (2.185, 3.975)	1.278 (1.063, 1.536)
Quartile 4	2.210 (1.492, 3.273)	2.879 (1.933, 4.289)	1.077 (0.861, 1.347)
*p* for linear trend ^e^	<0.001	<0.001	0.804

^a^ ORs and 95% CIs were estimated using logistic regression models. All models were adjusted for maternal age, maternal occupation status, paternal age, paternal smoking status, paternal alcohol consumption, gestational age, conception mode, fetus number, presence of risk factors, season of conception, and average temperature during corresponding period. ^b^ Embryonic period was defined as 3–8 weeks of gestation. ^c^ First trimester was defined as 1–13 weeks of gestation. ^d^ Preconception period was defined as 3 months before LMP. ^e^ *p* for linear trend was tested by including median of each quartile range as continuous variable in model.

## Data Availability

The data for O_3_ and PM_2.5_ can be found at http://tapdata.org.cn/ (accessed on 31 May 2024) and https://doi.org/10.5281/zenodo.3753614 (accessed on 31 May 2024), respectively. The clinical data are not publicly available.

## Supplementary Materials

The following supporting information can be downloaded at: https://www.mdpi.com/article/10.3390/toxics13060463/s1, Figure S1. Location of 1313 Maternal-Fetal Medicine centers in China. Figure S2. Flow chart of study participant selection. Table S1. The proportion of specific subtypes for CCHD. Table S2. Distributions of individual exposures to O_3_ during specific periods. Table S3. Association between maternal O_3_ exposure and CCHD, stratified by maternal, paternal, or clinical characteristics (per increase of 10 μg/m^3^ in O_3_). Table S4. Estimated OR (95% CI) of CCHD and its five most common subtypes related to each 10 μg/m^3^ increase in exposure to ambient O_3_. Table S5. Association of exposure to ambient O_3_ during periconception period and CCHD in China, 2013–2021. Table S6. Estimated ORs and 95% CIs of CCHD associated with ambient O_3_ exposure after adjustment of PM_2.5_ exposure and NO_2_ exposure during four periods in China, 2013–2021.

## References

[1] GBD 2017 Congenital Heart Disease Collaborators (2020). Global, regional, and national burden of congenital heart disease, 1990–2017: A systematic analysis for the Global Burden of Disease Study 2017. Lancet Child Adolesc. Health.

[2] Ossa Galvis M.M., Bhakta R.T., Tarmahomed A., Mendez M.D. (2025). Cyanotic Heart Disease. StatPearls [Internet].

[3] Lee K., Khoshnood B., Chen L., Wall S.N., Cromie W.J., Mittendorf R.L. (2001). Infant mortality from congenital malformations in the United States, 1970–1997. Obstet. Gynecol..

[4] Oster M.E., Lee K.A., Honein M.A., Riehle-Colarusso T., Shin M., Correa A. (2013). Temporal trends in survival among infants with critical congenital heart defects. Pediatrics.

[5] Su Z.H., Zou Z.Y., Hay S.I., Liu Y.W., Li S.J., Chen H.W., Mohsen N., Zimmerman M.S., Martin G.R., Wilner L.B. (2022). Global, regional, and national time trends in mortality for congenital heart disease, 1990–2019: An age-period-cohort analysis for the Global Burden of Disease 2019 study. EClinicalMedicine.

[6] Zhao Q.M., Liu F., Wu L., Ma X.J., Niu C., Huang G.Y. (2019). Prevalence of congenital heart disease at live birth in China. J. Pediatr..

[7] Martin G.R., Beekman R.H., Mikula E.B., Fasules J., Garg L.F., Kemper A.R., Morrow W.R., Pearson G.D., Mahle W.T. (2013). Implementing recommended screening for critical congenital heart disease. Pediatrics.

[8] American Institute of Ultrasound in Medicine (2013). AIUM practice guideline for the performance of fetal echocardiography. J. Ultrasound Med..

[9] Carvalho J.S., Allan L.D., Chaoui R., Copel J.A., DeVore G.R., Hecher K., Lee W., Munoz H., Paladini D., Tutschek B. (2013). ISUOG Practice Guidelines (updated): Sonographic screening examination of the fetal heart. Ultrasound Obstet. Gynecol..

[10] Tometzki A.J., Suda K., Kohl T., Kovalchin J.P., Silverman N.H. (1999). Accuracy of prenatal echocardiographic diagnosis and prognosis of fetuses with conotruncal anomalies. J. Am. Coll. Cardiol..

[11] Donofrio M.T., Moon-Grady A.J., Hornberger L.K., Copel J.A., Sklansky M.S., Abuhamad A., Cuneo B.F., Huhta J.C., Jonas R.A., Krishnan A. (2014). Diagnosis and treatment of fetal cardiac disease: A scientific statement from the American Heart Association. Circulation.

[12] Liberman R.F., Getz K.D., Lin A.E., Higgins C.A., Sekhavat S., Markenson G.R., Anderka M. (2014). Delayed diagnosis of critical congenital heart defects: Trends and associated factors. Pediatrics.

[13] Wadhwa P.D., Buss C., Entringer S., Swanson J.M. (2009). Developmental origins of health and disease: Brief history of the approach and current focus on epigenetic mechanisms. Semin. Reprod. Med..

[14] Wan X.Y., Wei S.X., Wang Y.Q., Jiang J., Lian X.Y., Zou Z.Y., Li J. (2023). The association between maternal air pollution exposure and the incidence of congenital heart diseases in children: A systematic review and meta-analysis. Sci. Total Environ..

[15] Sun H.Z., Zhao J., Liu X., Qiu M., Shen H., Guillas S., Giorio C., Staniaszek Z., Yu P., Wan M.W.L. (2023). Antagonism between ambient ozone increase and urbanization-oriented population migration on Chinese cardiopulmonary mortality. Innovation.

[16] Arjomandi M., Wong H., Donde A., Frelinger J., Dalton S., Ching W., Power K., Balmes J.R. (2015). Exposure to medium and high ambient levels of ozone causes adverse systemic inflammatory and cardiac autonomic effects. Am. J. Physiol. Heart Circ. Physiol..

[17] Day D.B., Xiang J., Mo J., Li F., Chung M., Gong J., Weschler C.J., Ohman-Strickland P.A., Sundell J., Weng W. (2017). Association of ozone exposure with cardiorespiratory pathophysiologic mechanisms in healthy adults. JAMA Intern. Med..

[18] Mirowsky J.E., Carraway M.S., Dhingra R., Tong H., Neas L., Diaz-Sanchez D., Cascio W., Case M., Crooks J., Hauser E.R. (2017). Ozone exposure is associated with acute changes in inflammation, fibrinolysis, and endothelial cell function in coronary artery disease patients. Environ. Health.

[19] Agay-Shay K., Friger M., Linn S., Peled A., Amitai Y., Peretz C. (2013). Air pollution and congenital heart defects. Environ. Res..

[20] Jiang W., Liu Z., Ni B., Xie W., Zhou H., Li X. (2021). Independent and interactive effects of air pollutants and ambient heat exposure on congenital heart defects. Reprod. Toxicol..

[21] Yang Y., Lin Q.M., Liang Y., Ruan Z.L., Acharya B.K., Zhang S.Y., Qian Z.M., McMillin S.E., Hinyard L., Sun J. (2020). Maternal air pollution exposure associated with risk of congenital heart defect in pre-pregnancy overweighted women. Sci. Total Environ..

[22] Zhang B., Zhao J.Z., Yang R., Qian Z.M., Liang S.W., Bassig B.A., Zhang Y.M., Hu K., Xu S.Q., Dong G.H. (2016). Ozone and other air pollutants and the risk of congenital heart defects. Sci. Rep..

[23] Dadvand P., Rankin J., Rushton S., Pless-Mulloli T. (2011). Ambient air pollution and congenital heart disease: A register-based study. Environ. Res..

[24] Farhi A., Boyko V., Almagor J., Benenson I., Segre E., Rudich Y., Stern E., Lerner-Geva L. (2014). The possible association between exposure to air pollution and the risk for congenital malformations. Environ. Res..

[25] Lavigne E., Lima I., Hatzopoulou M., Ryswyk K.V., Decou M.L., Luo W., Donkelaar A.V., Martin R.V., Chen H., Stieb D.M. (2019). Spatial variations in ambient ultrafine particle concentrations and risk of congenital heart defects. Environ. Int..

[26] Vinikoor-Imler L.C., Stewart T.G., Luben T.J., Davis J.A., Langlois P.H. (2015). An exploratory analysis of the relationship between ambient ozone and particulate matter concentrations during early pregnancy and selected birth defects in Texas. Environ. Pollut..

[27] Vrijheid M., Martinez D., Manzanares S., Dadvand P., Schembari A., Rankin J., Nieuwenhuijsen M. (2011). Ambient air pollution and risk of congenital anomalies: A systematic review and meta-analysis. Environ. Health Perspect..

[28] Yuan X.L., Liang F.C., Zhu J., Huang K.Y., Dai L., Li X.H., Wang Y.P., Li Q., Lu X.F., Huang J.F. (2023). Maternal Exposure to PM_2.5_ and the risk of congenital heart defects in 1.4 Million Births: A nationwide surveillance-based study. Circulation.

[29] Louis G.M., Cooney M.A., Lynch C.D., Handal A. (2008). Periconception window: Advising the pregnancy-planning couple. Fertil. Steril..

[30] Luderer U., Lim J.W., Ortiz L., Nguyen J.D., Shin J.H., Allen B.D., Liao L.S., Malott K., Perraud V., Wingen L.M. (2022). Exposure to environmentally relevant concentrations of ambient fine particulate matter (PM_2.5_) depletes the ovarian follicle reserve and causes sex-dependent cardiovascular changes in apolipoprotein E null mice. Part. Fibre Toxicol..

[31] Wu S.S., Zhang Y.S., Wu X.Q., Hao G.M., Ren H.Q., Qiu J.H., Zhang Y.F., Bi X.Y., Yang A.M., Bai L.N. (2021). Association between exposure to ambient air pollutants and the outcomes of in vitro fertilization treatment: A multicenter retrospective study. Environ. Int..

[32] Luoto R., Mottola M.F., Hilakivi-Clarke L. (2013). Pregnancy and lifestyle: Short- and long-term effects on mother’s and her children’s health. J. Pregnancy.

[33] Gao S., Han J.C., Yu S.M., Guo Y., Ruan Y.P., Fu Y.W., Hao X.Y., Wang X., Wang S.Y., Zhou X.X. (2021). Comparison of fetal echocardiogram with fetal cardiac autopsy findings in fetuses with congenital heart disease. J. Matern. Fetal Neonatal Med..

[34] Hoffman J.I., Kaplan S. (2002). The incidence of congenital heart disease. J. Am. Coll. Cardiol..

[35] Mai C.T., Riehle-Colarusso T., O’Halloran A., Cragan J.D., Olney R.S., Lin A., Feldkamp M., Botto L.D., Rickard R., Anderka M. (2012). Selected birth defects data from population-based birth defects surveillance programs in the United States, 2005–2009: Featuring critical congenital heart defects targeted for pulse oximetry screening. Birth Defects Res. A Clin. Mol. Teratol..

[36] Oster M.E., Aucott S.W., Glidewell J., Hackell J., Kochilas L., Martin G.R., Phillippi J.L., Pinto N.M., Saarinen A., Sontag M. (2016). Lessons learned from newborn screening for critical congenital heart defects. Pediatrics.

[37] Xiao Q., Geng G., Xue T., Liu S., Cai C., He K., Zhang Q. (2022). Tracking PM_2.5_ and O_3_ pollution and the related health burden in China 2013–2020. Environ. Sci. Technol..

[38] Hu C.Y., Huang K., Fang Y., Yang X.J., Ding K., Jiang W., Hua X.G., Huang D.Y., Jiang Z.X., Zhang X.J. (2020). Maternal air pollution exposure and congenital heart defects in offspring: A systematic review and meta-analysis. Chemosphere.

[39] Wei J., Li Z.Q., Cribb M., Huang W., Xue W.H., Sun L., Guo J.P., Peng Y.R., Li J., Lyapustin A. (2020). Improved 1km resolution PM_2.5_ estimates across China using enhanced space–time extremely randomized trees. Atmos. Chem. Phys..

[40] Wei J., Li Z., Lyapustin A., Sun L., Peng Y., Xue W., Su T.N., Cribb M. (2021). Reconstructing 1-km-resolution high-quality PM_2.5_ data records from 2000 to 2018 in China: Spatiotemporal variations and policy implications. Remote Sens. Environ..

[41] Wei J., Li Z., Wang J., Li C., Gupta P., Cribb M. (2023). Ground-level gaseous pollutants (NO_2_, SO_2_, and CO) in China: Daily seamless mapping and spatiotemporal variations. Atmos. Chem. Phys..

[42] Xin Y., Yang Y., Chen X., Yue X., Liu Y., Yin C. (2022). Evaluation of IMERG and ERA5 precipitation products over the Mongolian Plateau. Sci. Rep..

[43] R Core Team (2023). R: A Language and Environment for Statistical Computing. R Foundation for Statistical Computing. https://www.r-project.org/.

[44] Huang C.C., Chen B.Y., Pan S.C., Ho Y.L., Guo Y.L. (2019). Prenatal exposure to PM_2.5_ and Congenital Heart Diseases in Taiwan. Sci. Total Environ..

[45] Girguis M.S., Strickland M.J., Hu X., Liu Y., Bartell S.M., Vieira V.M. (2016). Maternal exposure to traffic-related air pollution and birth defects in Massachusetts. Environ. Res..

[46] Schembari A., Nieuwenhuijsen M.J., Salvador J., De Nazelle A., Cirach M., Dadvand P., Beelen R., Hoek G., Basagaña X., Vrijheid M. (2014). Traffic-related air pollution and congenital anomalies in Barcelona. Environ. Health Perspect..

[47] Wei J., Li Z., Pinker R.T., Wang J., Sun L., Xue W., Li R., Cribb M. (2021). Himawari-8-derived diurnal variations in ground-level PM_2.5_ pollution across China using the fast space-time Light Gradient Boosting Machine (LightGBM). Atmos. Chem. Phys..

[48] Ogliari K.S., Lichtenfels A.J., de Marchi M.R., Ferreira A.T., Dolhnikoff M., Saldiva P.H. (2013). Intrauterine exposure to diesel exhaust diminishes adult ovarian reserve. Fertil. Steril..

[49] Perin P.M., Maluf M., Januário D.N., Saldiva P.H. (2008). Effects of short-term exposure of female mice to diesel exhaust particles on in vitro fertilization and embryo development. Fertil. Steril..

[50] Klepac P., Locatelli I., Korošec S., Künzli N., Kukec A. (2018). Ambient air pollution and pregnancy outcomes: A comprehensive review and identification of environmental public health challenges. Environ. Res..

[51] Proietti E., Röösli M., Frey U., Latzin P. (2013). Air pollution during pregnancy and neonatal outcome: A review. J. Aerosol Med. Pulm. Drug Deliv..

[52] Januário D.A., Perin P.M., Maluf M., Lichtenfels A.J., Nascimento S.P.H. (2010). Biological effects and dose-response assessment of diesel exhaust particles on in vitro early embryo development in mice. Toxicol. Sci..

[53] Li Z., Tang Y., Song X., Lazar L., Li Z., Zhao J. (2019). Impact of ambient PM_2.5_ on adverse birth outcome and potential molecular mechanism. Ecotoxicol. Environ. Saf..

[54] Yauk C., Polyzos A., Rowan-Carroll A., Somers C.M., Godschalk R.W., Van Schooten F.J., Berndt M.L., Pogribny I.P., Koturbash I., Williams A. (2008). Germ-line mutations, DNA damage, and global hypermethylation in mice exposed to particulate air pollution in an urban/industrial location. Proc. Natl. Acad. Sci. USA.

[55] Blanc N., Liao J., Gilliland F., Zhang J.J., Berhane K., Huang G., Yan W., Chen Z. (2023). A systematic review of evidence for maternal preconception exposure to outdoor air pollution on Children’s health. Environ. Pollut..

