# Supplementary material for: Maternal Exposure to Ambient Ozone and Fetal Critical Congenital Heart Disease in China: A Large Multicenter Retrospective Cohort Study
Source: Toxics. 2025 May 31;13(6):463. doi: 10.3390/toxics13060463 (PMC12197276; doi:10.3390/toxics13060463)
Supplement: Supplementary file 1 [file toxics-13-00463-s001.zip › toxics-3627424-supplementary.pdf]

Supplemental materials

Maternal exposure to ambient ozone and fetal critical congenital heart disease in China: A large multicenter retrospective cohort study

Yanping Ruan[1#], Yaqi Wang[2 #], Zhiyong Zou[2*], Jing Li[2*] and Yihua He[1*]

[#]These authors contributed equally to this work.

Affiliations:

[1]Department of Echocardiography, Maternal-Fetal Medicine Research Consultation Center, Beijing Anzhen Hospital, Capital Medical University, Beijing, 100029, China.

[2]Institute of Child and Adolescent Health, School of Public Health, Peking University; National Health Commission Key Laboratory of Reproductive Health, Beijing 100191, China.

***Correspondence:**

Yihua He − Department of Echocardiography, Maternal-Fetal Medicine Research Consultation Center, Beijing Anzhen Hospital, Capital Medical University, Beijing, 100029, China. Email: heyihuaecho@hotmail.com.

Jing Li − Institute of Child and Adolescent Health, School of Public Health, Peking University; National Health Commission Key Laboratory of Reproductive Health, Beijing 100191, China. Email: jing.li@hsc.pku.edu.cn.

Zhiyong Zou − Institute of Child and Adolescent Health, School of Public Health, Peking University; National Health Commission Key Laboratory of Reproductive Health, Beijing 100191, China. Email: harveyzou2002@bjmu.edu.cn.

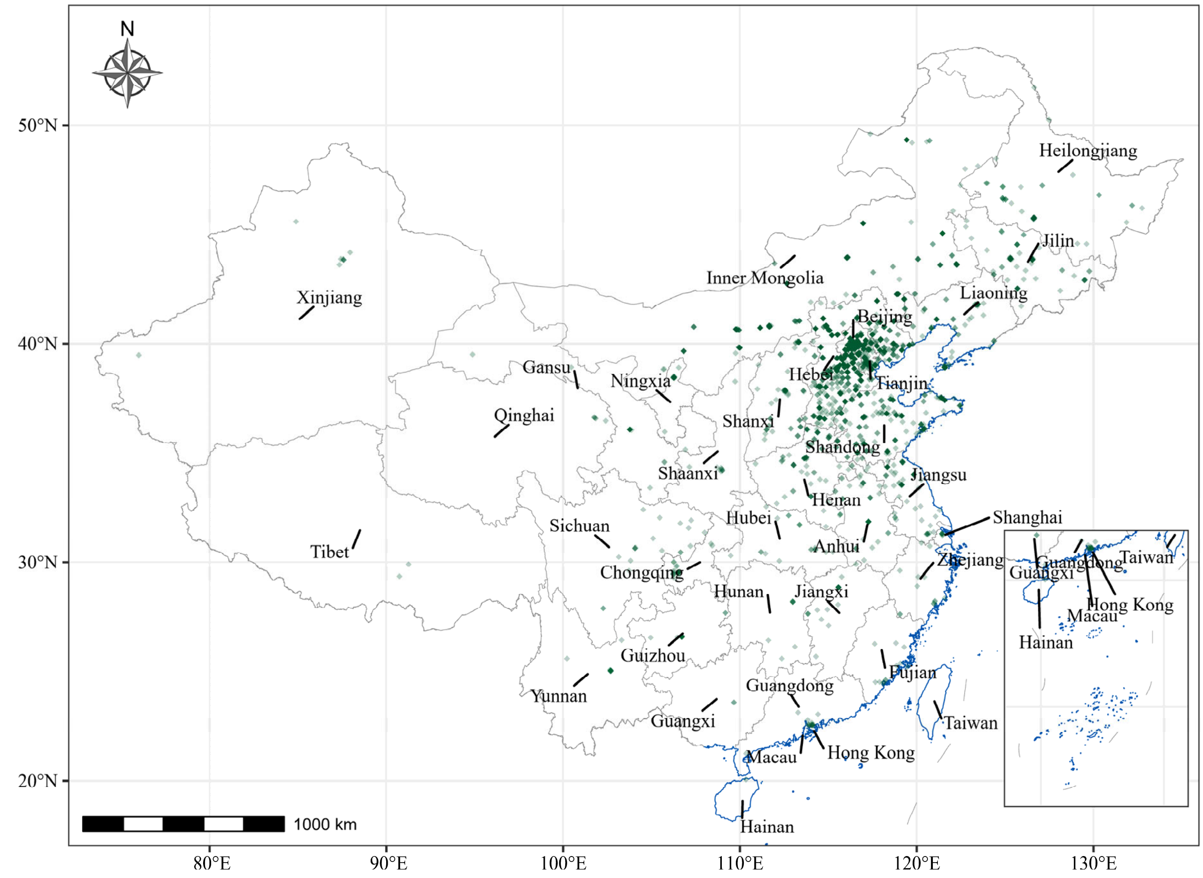

Figure S1. Location of 1313 Maternal-Fetal Medicine centers in China. The green diamond represents the address of the hospital.

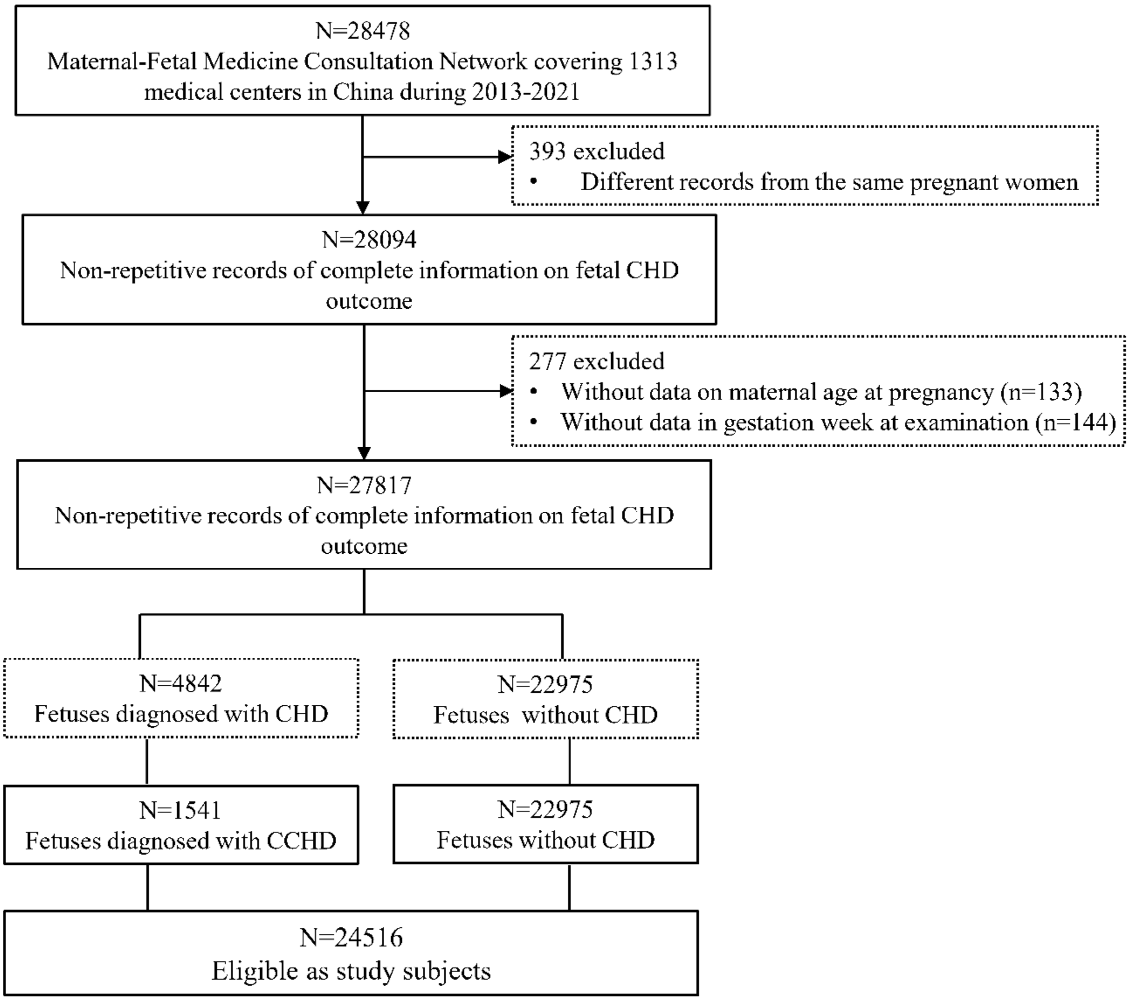

Figure S2 Flow chart of study participant selection. CHD, congenital heart defects. CCHD, critical congenital heart defects.

Table S1. The proportion of specific subtypes for CCHD.

Types of CCHD	Number (%)
**Total**	1541 (100%)
**Tetralogy of Fallot**	426 (27.6%)
**Coarctation of the aorta**	250 (16.2%)
**Double-outlet right ventricle**	231 (15.0%)
**D-transposition of the great arteries**	163 (10.6%)
**Total anomalous pulmonary venous connection**	99 (6.4%)
**Interrupted aortic arch**	82 (5.3%)
**Hypoplastic left heart syndrome**	78 (5.1%)
**Single ventricle**	77 (5.0%)
**Pulmonary atresia with intact septum**	74 (4.8%)
**Tricuspid atresia**	26 (1.7%)
**Truncus arteriosus**	52 (3.4%)
**Ebstein anomaly**	47 (3.0%)
**Two or more types**	64 (4.1%)

CCHD, critical congenital heart defects.

Table S2. Distributions of individual exposures to O_3 during specific periods.

Period	Pollutant	Mean ± SD	Min	P_{25}	Median	P_{75}	Max
Periconception period[a]	O_3, μg/m^3	93.9±29.9	38.1	65.8	91.3	121.8	161.6
	Temperature (°K)	285.6±7.5	261.6	278.2	285.4	293.0	300.1
	Relative humidity (%)	49.8±8.8	28.7	43.4	49.2	55.8	87.1
Embryonic period[b]	O_3, μg/m^3	95.6±43.3	18.9	56.1	91.7	134.7	205.6
	Temperature (°K)	285.9±10.9	252.7	275.2	286.9	296.7	303.8
	Relative humidity (%)	50.3±13.3	21.5	38.3	47.7	61.4	88.8
The first trimester[c]	O_3, μg/m^3	95.9±40.2	22.0	59.2	91.9	132.2	188.0
	Temperature (°K)	286.0±10.2	254.7	276.4	286.7	295.9	302.5
	Relative humidity (%)	50.1±11.8	25.3	40.0	48.0	60.6	88.2
Preconception period[d]	O_3, μg/m^3	92.0±39.4	22.8	55.1	88.6	127.7	185.2
	Temperature (°K)	285.3±10.0	254.6	275.5	285.6	294.9	302.1
	Relative humidity (%)	49.6±11.4	23.7	40.2	47.3	59.7	88.1

Abbreviations: O_3, ozone; P_{25}, the 25th percentile; P_{75}, the 75th percentile; SD, standard error.

[a] Periconception period was defined as 3 months before LMP until 13 weeks into pregnancy.

[b] Embryonic period was defined as 3-8 weeks of gestation.

[c] The first trimester was defined as 1-13 weeks of gestation.

[d] Preconception period was defined as 3 months before LMP.

Table S3. Estimated OR (95% CI) of CCHD associated with each 10 μg/m^3 increase in exposure to ambient O_3 stratified by maternal, paternal or clinical characteristics.

Stratified factors	Exposure Windows		
	Periconception period	Embryonic period	The first trimester
**Maternal age**			
<35 years	1.125 (1.069, 1.183)	1.145 (1.094, 1.198)	1.182 (1.123, 1.244)
≥35 years	1.055 (0.945, 1.178)	1.095 (0.990, 1.210)	1.184 (1.057, 1.326)
P for effect modification[b]	0.171	0.782	0.953
**Paternal age**			
<35 years	1.156 (1.095, 1.220)	1.149 (1.096, 1.206)	1.187 (1.125, 1.252)
≥35 years	1.004 (0.916, 1.100)	1.103 (1.017, 1.196)	1.175 (1.072, 1.288)
P for effect modification[b]	0.612	0.444	0.249
**Maternal Occupation**			
Employed	1.106 (1.047, 1.167)	1.122 (1.068, 1.178)	1.175 (1.112, 1.242)
Unemployed	1.151 (1.052, 1.260)	1.169 (1.080, 1.266)	1.200 (1.098, 1.312)
P for effect modification[b]	0.317	0.414	0.383
**Paternal smoking**			
Smoking or ever smoked	1.167 (1.019, 1.336)	1.137 (1.012, 1.278)	1.170 (1.028, 1.332)
Never	1.109 (1.056, 1.165)	1.139 (1.089, 1.190)	1.188 (1.130, 1.248)
P for effect modification[b]	0.436	0.669	0.744
**Presence of risk factor[a]**			
Yes	1.125 (1.067, 1.187)	1.138 (1.084, 1.195)	1.190 (1.126, 1.258)
No	1.057 (0.962, 1.161)	1.124 (1.039, 1.216)	1.158 (1.060, 1.263)
P for effect modification[b]	0.247	0.203	0.093
**Conception mode**			
Natural	1.115 (1.064, 1.169)	1.135 (1.088, 1.184)	1.178 (1.124, 1.235)
IVF	1.128 (0.867, 1.466)	1.153 (0.907, 1.464)	1.259 (0.959, 1.654)
P for effect modification[b]	0.416	0.972	0.808

CCHD, critical congenital heart defect; OR, odds ratio; O_3, ozone.

[a] Risk factors included the following information: commodities (diabetes mellitus, gestational diabetes mellitus, upper respiratory infections within 3 months before conception, anemia during the first trimester, phenylketonuria, metabolic disorders, thyroid disease, and connective tissue diseases), history of CHD, adverse results of prenatal genetic test results, radioactive substances exposure, mental stress, household environment (Keeping pets, decoration) during the early pregnancy. The presence of any aforementioned history was defined as having risk factors.

[b] *P* for effect modification was estimated using likelihood ratio test by comparing logistic regression models with or without an interaction term of O_3 and each stratification variable.

Table S4. Estimated OR (95% CI) of CCHD and its five most common subtypes associated with each 10 μg/m³ increase in exposure to ambient O_3.

CCHD Subtypes	ORs (95% CIs)[a]			
	**Periconception period[b]**	**Embryonic period[c]**	**The first trimester[d]**	**Preconception period[e]**
**TOF**	0.978 (0.876, 1.091)	1.178 (1.084, 1.281)	1.123 (1.075, 1.174)	1.186 (1.068, 1.317)
**COA**	0.785 (0.688, 1.096)	1.036 (0.932, 1.152)	1.079 (1.020, 1.1420)	1.116 (0.977, 1.274)
**DORV**	0.784 (0.684, 1.100)	0.992 (0.886, 1.112)	1.107 (1.044, 1.175)	1.050 (0.916, 1.203)
**TGA**	0.914 (0.758, 1.103)	1.240 (1.071, 1.436)	1.258 (1.159, 1.366)	1.218 (1.019, 1.456)
**TAPVC**	0.936 (0.751, 1.166)	1.198 (1.007, 1.426)	1.143 (1.048, 1.247)	1.240 (1.004, 1.530)

TOF, Tetralogy of Fallot; COA, Coarctation of the aorta; DORV; Double-outlet right ventricle. TGA, D-transposition of the great arteries; TAPVC, Total anomalous pulmonary venous connection.

[a] ORs and 95% CIs were estimated using logistic regression models. All models were adjusted for maternal age, maternal occupation status, paternal age, paternal smoking status, paternal alcohol consumption, gestational age, conception mode, fetus number, presence of risk factors, season of conception, $PM_{2.5}$ exposure, average temperature (natural cubic splines with 3 degrees of freedom [df]) and relative humidity (natural cubic splines with 3 degrees of freedom [df]) during corresponding period.

[b] Periconception period was defined as 3 months before LMP until 13 weeks of gestation.

[c] Embryonic period was defined as 3-8 weeks of gestation.

[d] The first trimester was defined as 1-13 weeks of gestation.

[e] Preconception period was defined as 3 months before LMP.

Table S5. Association of exposure to ambient O_3 during periconception period[a] and CCHD in China, 2013-2021.

O_3 exposure	ORs (95% CIs)[b]	
	**Model 1[c]**	**Model 2[d]**
Per 10 ug/m^3	1.406 (1.330, 1.486)	1.422 (1.345, 1.503)
Quartile 1	Ref	Ref
Quartile 2	1.549 (1.317, 1.821)	1.570 (1.335, 1.847)
Quartile 3	2.465 (2.056, 2.956)	2.471 (2.059, 2.964)
Quartile 4	3.157 (2.462, 4.048)	3.240 (2.524, 4.158)
P for linear trend[e]	<0.001	<0.001

CCHD, critical congenital heart defects; O_3, ozone; OR, odds ratio; CI, confidence interval.

[a]Periconception period was defined as 3 months before LMP until 13 weeks of gestation.

[b]ORs and 95% CIs were estimated using logistic regression models.

[c]Model 1: Adjusted for maternal age, maternal occupation status, paternal age, average temperature (natural cubic splines with 3 degrees of freedom [df]) and relative humidity (natural cubic splines with 3 df).

[d]Model 2: Model 1 plus paternal smoking status and paternal alcohol consumption.

[e]P for linear trend was tested by including the median of each quartile range as a continuous variable in the model.

Table S6. Estimated ORs and 95% CIs[a] of CCHD associated with ambient O_3 exposure after adjustment of $PM_{2.5}$ exposure and NO_2 exposure during four periods in China, 2013-2021.

O_3 exposure	OR (95% CI)[a]	
	With adjustment for $PM_{2.5}$ exposure	With adjustment for NO_2 exposure
**Embryonic period[b]**	1.127 (1.076, 1.180)	1.077 (1.022, 1.135)
**The first trimester[c]**	1.196 (1.133, 1.261)	1.126 (1.060, 1.197)
**Preconception period[d]**	1.203 (1.136, 1.274)	1.095 (1.028, 1.167)

CCHD, critical congenital heart defects; NO_2, nitrogen dioxide; O_3, Ozone; $PM_{2.5}$, particulate Matter $\leqslant$2.5 micrometers or less in diameter; OR, odds ratio; CI, confidence interval.

[a] ORs and 95% CIs were estimated using logistic regression models. All models were adjusted for maternal age, maternal occupation status, paternal age, paternal smoking status, paternal alcohol consumption, gestational age, conception mode, fetus number, presence of risk factors, season of conception, $PM_{2.5}$ exposure, average temperature (natural cubic splines with 3 degrees of freedom [df]) and relative humidity (natural cubic splines with 3 degrees of freedom [df]) during corresponding period.

[b] Embryonic period was defined as 3-8 weeks of gestation.

[c] The first trimester was defined as 1-13 weeks of gestation.

[d] Preconception period was defined as 3 months before LMP.
